# Fluid-Structure Interaction Analysis on the Influence of the Aortic Valve Stent Leaflet Structure in Hemodynamics

**DOI:** 10.3389/fphys.2022.904453

**Published:** 2022-05-13

**Authors:** Xiangkun Liu, Wen Zhang, Ping Ye, Qiyi Luo, Zhaohua Chang

**Affiliations:** School of Health Science and Engineering, University of Shanghai for Science and Technology, Shanghai, China

**Keywords:** aortic valve stent, leaflet structure, fluid-structure interaction, computational fluid dynamics, operator-split lagrangian eulerian, hemodynamic effect

## Abstract

Transcatheter aortic valve replacement (TAVR) is a minimally invasive surgical treatment for heart valve disease. At present, personalized TAVR valves are not available for some patients. This study adopts the fluid-structure interaction (FSI) model of the research object that has a three-disc leaflet form and structural design in the valve leaflet area. The valve opening shape, orifice area, stress-strain, and distribution of hemodynamic flow and pressure were compared under the condition of equal contact area between valve and blood. The FSI method was used to simulate the complex three dimensional characteristics of the flow field more accurately around the valve after TAVR stent implantation. Three personalized stent systems were established to study the performance of the leaflet design based on computational fluid dynamics. By comparing the different leaflet geometries, the maximum stress on leaflets and stents of model B was relatively reduced, which effectively improved the reliability of the stent design. Such valve design also causes the opening area of the valve leaflet to increase and the low-velocity area of the flow field to decrease during the working process of the valve, thus reducing the possibility of thrombosis. These findings can underpin breakthroughs in product design, and provide important theoretical support and technical guidance for clinical research.

## Introduction

Among cardiovascular diseases, aortic valve disease has the second highest morbidity and mortality in the world (Go et al., 2013). Nearly 30,000 patients worldwide undergo aortic valve-replacement surgery every year (Dasi et al., 2009). Transcatheter aortic valves (TAVs) were initially used as a minimally invasive alternative to thoracotomy in order to replace the aortic valve (Guidoin et al., 2010), and mainly used in patients that had high surgical risk. In recent years, due to the advancement of related technologies and the development of clinical treatments, the use of TAVs has gradually expanded to reach low- and moderate-risk patients. Numerical simulation can provide detailed information that is difficult to obtain from experiments, and can help to evaluate the impact of blood flow on valve biomechanics. Such findings, in turn, can be used to guide clinical interventions for the treatment of aortic valve disease. Due to the strong interaction between the aortic valve and the surrounding blood, fluid-structure interaction (FSI) analysis is widely used and is considered the best method of numerical simulation for accurate simulation of the valve load and the surrounding flow field (Luraghi et al., 2021).

Transcatheter aortic valve (TAV) devices consist of three biological valve leaflets, self-expanding or mechanically expandable metal stents, and inner or outer skirts (Wei et al., 2018). This arrangement differs from traditional thoracotomy replacement valves and brings new challenges to FSI simulation. Wu et al. (2016) used the immersed boundary (IB) method to conduct an FSI simulation of a self-expanding TAV for the first time (Rotman et al., 2018). Subsequently, quite a few researchers have been focused on developing the accuracy and validity of the FSI method for transcatheter aortic valve replacement (TAVR) simulation. Both moving-mesh methods such as the arbitrary Lagrangian-Eulerian method (Ghosh et al., 2018) and fixed-mesh methods such as the immersed boundary method, and even combined fixed-moving grid methods such as the “operator-split” Lagrangian-Eulerian method (Luraghi et al., 2019) and mesh-free method such as the smoothed particle hemodynamic method (Pasta et al., 2020), have been applied. On the other hand, although TAVR complications have been decreasing since its introduction, some adverse outcomes are still present including leaflet durability, paravalvular leaks, and thrombosis (Luraghi et al., 2021), which makes the mechanical and hemodynamic performance of the TAV device of great concern. With a patient-specific model or an ideal model, FSI simulations have been performed to evaluate the leaflet opening area (Wu et al., 2016), mechanical stress (Ghosh et al., 2020), wall shear stress (Kandail et al., 2018), PVL severity (Luraghi et al., 2019), and the influence of calcification (Luraghi et al., 2020) or calcification of the bicuspid native aortic valve (Pasta et al., 2020), and so on.

However, most studies only involve one or two particular TAV devices. When it comes to the problem of understanding how the design parameters of a TAV device affect its performance, the relevant literature, to the best of our knowledge, is limited. Van Aswegen et al. (2012) modified a prosthetic aortic valve and created two configurations of the attachment to the surrounding stent. Through FSI simulation, the von Mises stress distribution was shown to be different between two configurations. However, the aortic root model used was highly simplified. Travaglino et al. (2020) parametrized a generic TAV model and developed a Bayesian optimization approach that succeeded in reducing the peak stress under a blood pressure of 120 mmHg. In their study, however, only leaflets were considered and finite element analysis was applied instead of FSI simulation. Carbonaro et al. (2021) utilized a mesh-morphing procedure to parametrize the TAV frame, and finite element analyses of TAV implantation were performed in idealized aortic root models with and without calcification. A multi-objective design optimization was conducted by coupling the design of the experiment with surrogate modeling to optimize the magnitude of the pullout force, peak maximum principal stress within the aortic wall, and contact pressure in the left ventricular outflow tract. Again, finite element analysis was applied instead of FSI simulation, and leaflet geometry was neglected. Thus, the influence of different design parameters needs to be further studied with a more complete TAV geometry model, and with consideration of the interaction between the device and blood.

In terms of the research literature concerning valve support, some researchers typically use numerical methods to evaluate the accuracy and validity of the heart valve unit (De Hart et al., 2003; Ghosh et al., 2018; Wu et al., 2019; Luraghi et al., 2021), while others study the performance of heart valve devices already on the market (Luraghi et al., 2019; Pasta et al., 2020; Pasta and Gandolfo, 2021). Most of these studies do not involve basic design methods, especially the relationship between structural design and flow fields, as well as related parametric studies. The main purpose of the current paper is to study how different flap designs affect the mechanical properties and flow field of TAV using the FSI method based on computational fluid dynamics, which would be instructive to TAV designing and clinical practice in terms of improving product performance. With the TAV device deployed in an ideal aortic root model, three parametrically modified leaflet designs with the same contact area with blood were investigated. Through computational fluid dynamics, intravascular hemodynamic characteristic, including the blood flow velocity and pressure distribution were assessed after the implantation of each valve stent. Using numerical calculations and comparisons, it was found that different valve shapes have a great impact on the valve opening area, stent force, and intravascular flow field.

## Methods

### Aortic Valve Stents Geometry

Aortic valve stents typically consist of three leaflets, self- or mechanically expandable stents, and inner/outer skirts (Wei et al., 2018). The stent is divided into two parts: the inflow tract and the outflow tract. The three leaflets and the skirt are sutured in the inflow tract area of the stent. In the present study, three valve leaflet geometries were designed for the same stent, and three personalized heart valve stent devices were established. Twelve basic units were arranged in the circumferential direction of the stent and 2.5 basic units were arranged in the axial direction of the stent. According to the specific aortic root structure of the patient, the axial length of the stent was controlled at about 30 mm, the initial stent radius diameter was *R* = 3.5 mm, the number of basic circumferential units was *n*_*c*_ = 12, the number of axial basic units was *n*_*a*_ = 2.5, the wall thickness was *t* = 0.42 mm, the trunk width was *t*_*b*_ = 0.614 mm, the branch trunk width was *t*_*a*_ = 0.3 mm, the inner arc radius was *r*_*a*_ = 0.07 mm, and *r*_*b*_ = 1 mm. The single cell width was calculated as:

$$w=2\pi R/n_{c}$$ (1)

**FIGURE 1:**
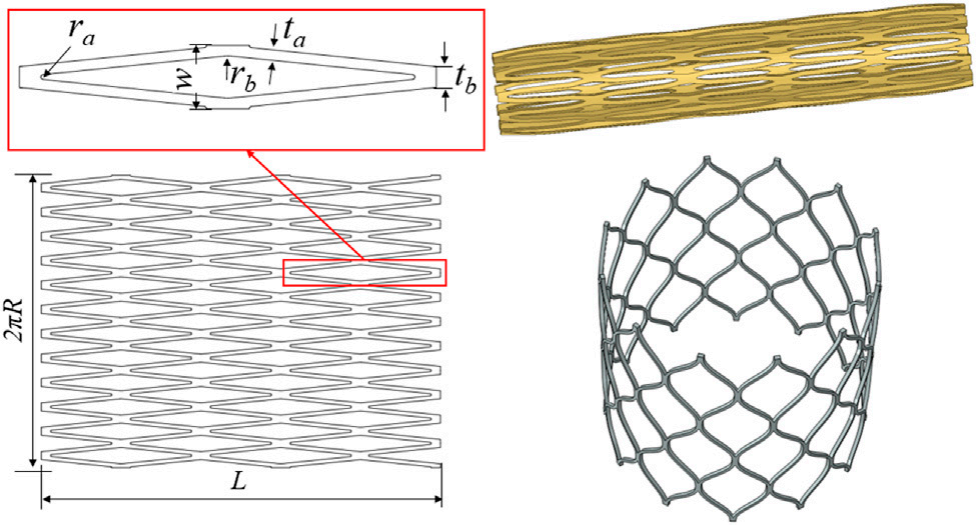
Diagram of the geometric structure of the aortic valve stent and three-dimensional model.

The original tube diameter of the stent was 7.0 mm, the wall thickness was 0.4 mm, and the expanded diameter of the stent was 27 mm. Figure 1 shows the geometric structure of the aortic valve stent and the three-dimensional model used in this study. A complete parametric CAD model of the stent was established using SolidWorks (Dassault Systèmes SolidWorks Corp., Waltham, MA, United States). For the mesh generation of the stent, the unit size of the stent model was controlled at 0.1 mm, the number of self-expanding stent units was 17,280, and the number of nodes was 37,440. Abaqus (SIMULIA, Johnston, RI, United States) was used to complete the processing of the stent model. First, according to the design parameters of the stent, a two-dimensional plane model of the stent was established, and the mesh division was completed. Second, the mesh model was wrapped so as to form a tubular shape to establish a laser cutting stent model, the diameter of the stent being 7.0 mm. Third, the laser cutting stent model was expanded and finalized. In other words, the process was divided into three steps: the diameter of the stent was expanded from 7 to 12 mm, then to 19 mm, and then to 27 mm, which completed the expansion of the stent.

With reference to the waveform structure of the stent, three structural forms of the valve were established to compare mechanical properties. The geometric design of the valve refers to the aortic structure proposed by De Hart et al. (2003) and was drawn on the connecting line with the stent commissure using reference points. The physiological structure of the aortic geometry shows three main components: the root, the base, and the tubular ascending aorta. The bases of the three aortic valve leaflets followed a hyperbola from one connection point to another at the aortic root. The aortic root began to form the sinus cavity, which was the origin of the ascending aorta. The three sinus cavities consisted of three circular arcs forming a clover-shaped section with α angles of 60°. Both the bottom part and the ascending part of the aorta were composed of cylinders.

**FIGURE 2:**
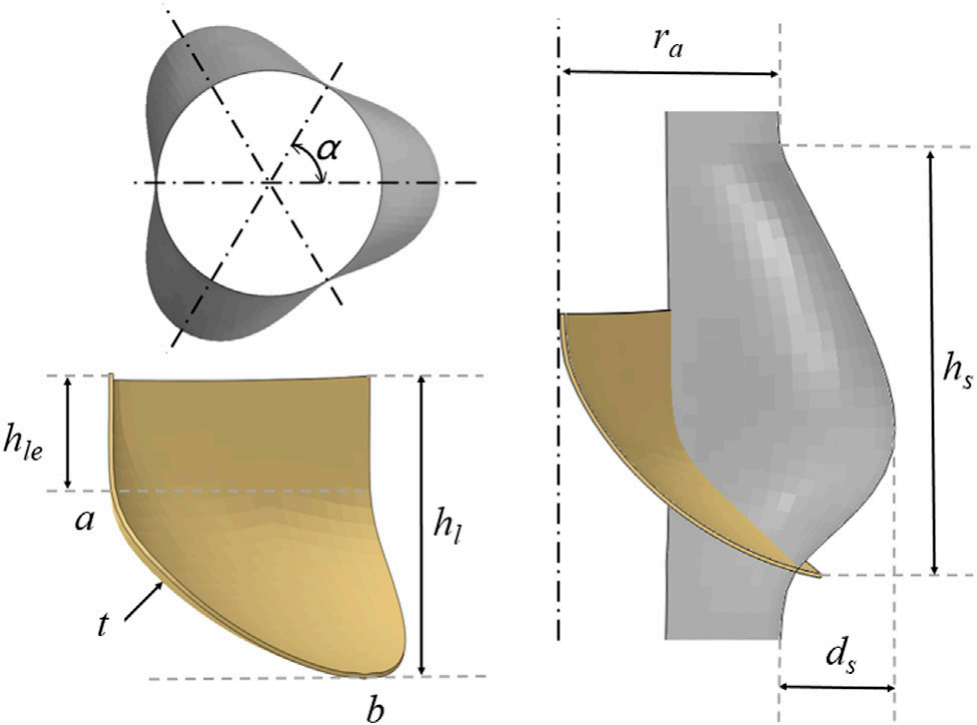
Schematic diagram of the geometry of the aorta and valve leaflets and definition of relevant dimensions of the aorta and valve leaflets.

Figure 2 shows the relevant dimensions of the aortic root geometry and valve leaflet geometry, as well as the established aortic wall model and the valve leaflet model based on the dimensions of the physiological structure shown in Tables 1, 2. In this study, *r*_*a*_ is the aortic valve radius, *d*_*s*_ is the sinus depth, *h*_*s*_ is the sinus height, *h*_*1*_ is the total leaflet height thickness, *h*_*1e*_ is the vertical leaflet height thickness, *t* is the leaflet thickness, and *Line* is the spatial location of the leaflet curve from point *a* to point *b*. Afterwards, the spatial position of the *Line* was determined, which can generate the valve. During modeling of the valve, the position of point *a* is defined at the coordinate origin. The CAD models of the leaflets and skirt were established using SolidWorks. For the mesh generation of valve leaflet and skirt, the number of three leaflet elements was 16,080 and the number of nodes was 24,885; the number of skirt elements was 25,176 and the number of nodes was 40,358. Size parameters for the aortic model structure and valve leaflets model are shown in Tables 1, 2.

**TABLE 1:** Aortic model structure size parameters (mm).

	*r*_*a*_	*d*_*s*_	*h*_*s*_	*α*
Aortic model	11.40	5.68	21.00	60°

**TABLE 2:** Valve leaflets model structure size parameters (mm).

	*h*_*1*_	*h*_*1e*_	*Line*
Model A	13.0	4.2	−4.243*ln(x)* + 8.1557
Model B	14.0	3.5	−4.727*ln(x)* + 10.867
Model C	16.0	3.2	−5.492*ln(x)* + 13.229

**FIGURE 3:**
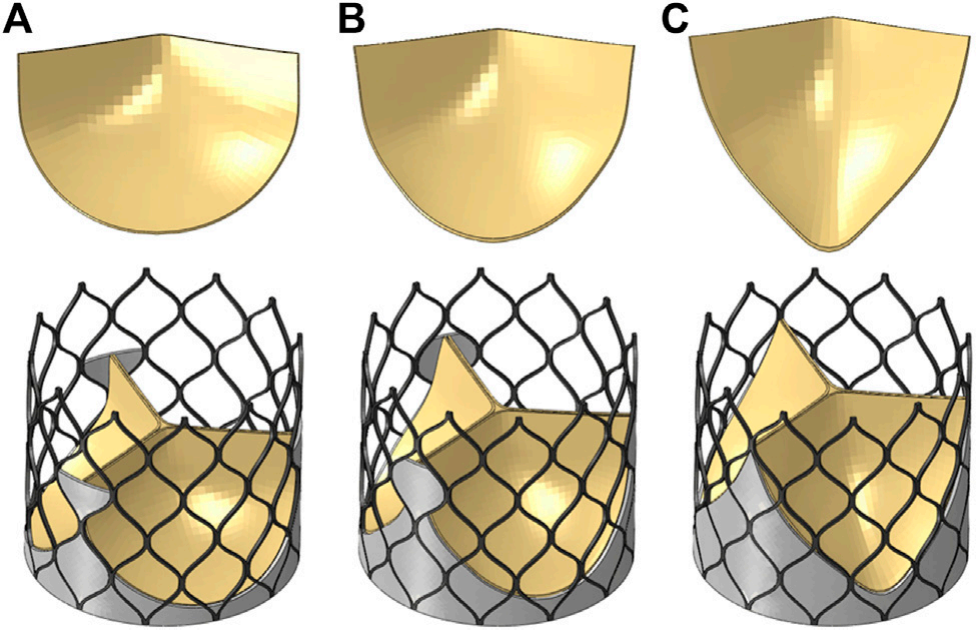
Three valve leaflet models and three valve stent models, **(A)** model A, **(B)** model B, **(C)** model C.

The valve leaflet model based on the size of the physiological structure had an area of about 350 mm^2^. When establishing the other two valve models, the valve and blood contact area of the three models was the same. These valve models ware established with reference to the stent design, and the valve area was equal in the three structures. The leaflet thickness was *t* = 0.2 mm. Model A was based on the size of the physiological structure. The upper half of the valve leaflet fell along the axis of the stent rod unit, and the lower half related to the shape of the bottom of the aortic sinus cavity. The bottom line of the leaflet of model B was basically perpendicular to the axis of the stent rod or intersected it at a certain angle. The bottom line of the leaflet of model C was along the axis of the stent rod, which was basically consistent with the axis. A complete stent model with three leaflet shapes was established (Figure 3). Because the skirt structure played a role in preventing peripheral leakage and was close to the aortic wall after implantation, it had little effect on the central flow field. The structure was simplified and was equal to the thickness of the stent, and the inner skirt coincided with the bottom edge of the valve leaflet. After the position of *Line* was determined, the valve leaflet, skirt, and stent were integrated to generate the aortic valve device. The integration of valve leaflet, skirt, and stent was completed through the fusion and sharing of near nodes. Sewing sutures among the stent, leaflets, and skirt was neglected.

### Valve Stent Implantation

**FIGURE 4:**
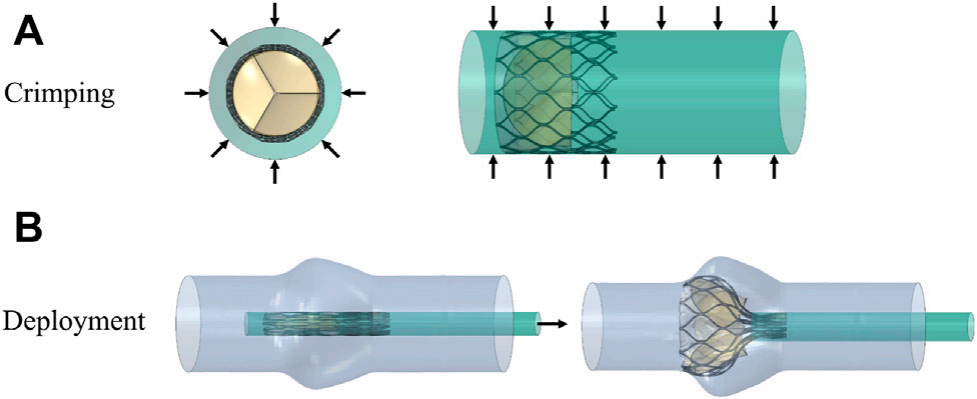
Schematic diagram of the valve stent intervention process, **(A)** stent crimping by applying a radial displacement on an outer cylindrical surface and **(B)** progressive deployment of the crimped stent.

The valve stent was delivered to the aortic root region by a delivery system and self-expanding to a predetermined position to play a supporting role. Combining stent production and processing technology, a three-dimensional model of the valve stent was developed using finite element simulation, and hemodynamic fluid-structure coupling analysis of the valve stent was carried out. Abaqus was used to simulate valve stent implantation, which included the crimp and self-expanding release of TAV device. In the first step, TAV device crimp analysis was performed, using a crimping tool to crimp the valve stent from a diameter of 27 to 10 mm. The crimping tool was used instead of a rigid cylinder surface. In the cylindrical coordinate system, radial displacement boundary conditions were applied to the rigid cylinder surface to gradually shrink it radially so as to crimp the stent, as shown in Figure 4. The contact between the inner surface of the rigid cylinder and the outer surface of the stent was defined as face-to-face, where the inner surface of the rigid cylinder surface was the main surface and the friction coefficient was defined as 0.1.

**FIGURE 5:**
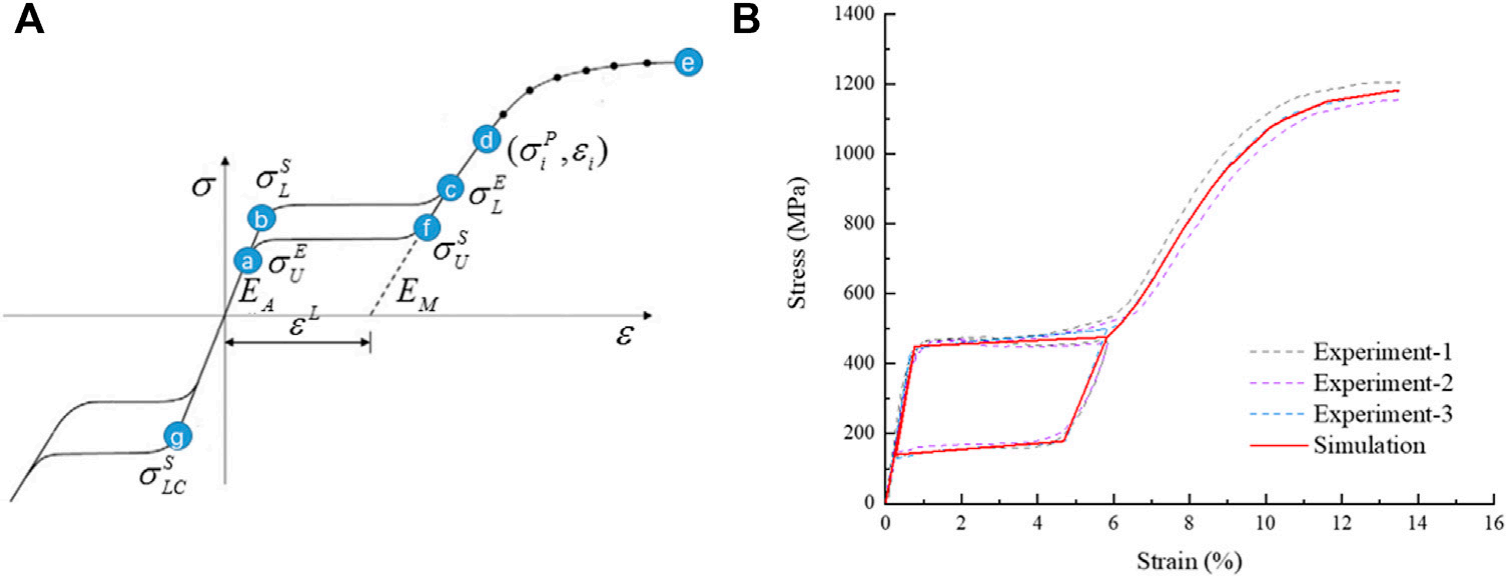
Schematic diagram of NiTi material properties, **(A)** the tensile strain-stress curve of the material and **(B)** the stress-strain curve fitted by simulation analysis.

The material of the stent was a nickel-titanium alloy, a hyperelastic material with coupled temperature parameters and mechanical parameters. This material model divided strain into three components: elastic strain, phase transformation strain, and plastic strain. When the material was completely transformed into martensite, the strain became plastic strain. The material data required for the simulation analysis were obtained with uniaxial tensile tests from loading and unloading, and reverse loading and unloading. Uniaxial tensile tests were conducted with an Instron 5,565 tensile tester (Instron Corporation, Norwood, MA, United States) at 37 ± 0.2°C to obtain stress-strain data. In the first group, the nickel-titanium wires were stretched to a strain of 6% then released; the other group of nickel-titanium wires were stretched to a strain of about 13.5%, and all the test data were recorded. The experimental data were fitted to a stress-strain curve, and the curve was used for simulation analysis as shown in Figure 5. The material property parameters were: E_A_ = 59,000 MPa, martensitic elastic modulus E_M_ = 26,100 MPa, Poisson’s ratio = 0.33, and the tensile limit of the material = 13.5%.

In the second step, TAV device was released and bounced back. In the cylindrical coordinate system, TAV device was released after being crimped and held by the crimping tool at the aortic valve site, and release analysis of TAV device in the aortic valve was conducted. During the entire analysis, the penalty function of self-contact was defined in the analysis. The face-to-face contact between TAV device and the aortic valve was defined as a penalty function contact, and the friction coefficient was set as 0.2.

**FIGURE 6:**
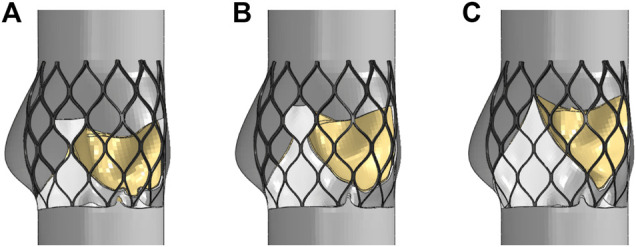
Structural model after valve stent intervention, **(A)** valve stent intervention of model A, **(B)** valve stent intervention of model B, **(C)** valve stent intervention of model C.

The leaflets and skirts were modeled as linear elastic materials with a Young’s modulus of 1 Mpa, a Poisson’s ratio of 0.45, and a density of 1,100 kg/m^3^ (Luraghi et al., 2019). The penalty function of self-contact was defined in the analysis. The suture between the skirt and the stent, and the suture between the leaflet and the skirt were approximated as a bound contact; that is, the edge of the skirt was bound to the inflow end of the stent, and the edge of the three leaflets was bound to the skirt. A simplified model of the aorta with three stents that was solved by Abaqus is shown in Figure 6. The upper and lower ends of the stent had a slight tendency to buckle inward, and the circumferential shape changed with the structure of the aortic sinus cavity. Because the areas of the three valve leaflet models were the same, the valve leaflet height gradually approached the sinus height.

In this study, Abaqus was applied to complete the modeling and finalization of the stent. The stent was implanted into the ideal heart model to obtain a simulation model of the stent when implanted. The simulation model completed in Abaqus was substituted into LS-DYNA (ANSYS, Canonsburg, PA, United States), and fluid-structure coupling related simulation analysis was carried out in LS-DYNA. In general, the molding and implantation of the stent were preliminarily completed in Abaqus software, and the fluid-structure coupling study was carried out and post-processed in LS-DYNA.

### Fluid-Structure Interaction Analysis

In a CFD only analysis, the moving reference frame was fixed in space, and a full Eulerian formulation was achieved in the LS-DYNA software. However, in cases of problems regarding fluid-structure interaction (FSI), the boundaries between the solid and fluid are Lagrangian and deform with the structure. An arbitrary Lagrangian-Eulerian (ALE) formulation was therefore retrieved. This approach allowed a strong and exact imposition of the solid boundary conditions on the fluid. The solid and fluid geometry must match at the interface but not necessarily the meshes. For FSI simulations, the solver used an ALE approach for mesh movement, which means that large deformations of the fluid mesh could occur. By default, the solver only rebuilt the mesh if elements got inverted. An “operator-split” Lagrangian-Eulerian method (Marom, 2015) was adopted using the finite element software LS-DYNA. The structure was handled in a Lagrangian manner, while the calculation of the Eulerian fluid conservation equations was split into two steps. In the first Lagrangian step, the mesh moved with fluid particles and the following mass conservation equation (Marom, 2015) and the Navier-Stokes equation (Luraghi et al., 2019) were solved:

$$\begin{array}{c} \rho J=\rho_{0} \end{array}$$ (2)

$$\begin{array}{c} \rho \frac{\partial v_{i}}{\partial t}+\rho v_{i}\frac{\partial v_{j}}{\partial x_{j}}=\frac{\partial \sigma_{ij}}{\partial x_{j}}+\rho f_{i} \end{array}$$ (3)

Where ρ is the density of the fluid, J the volumetric strain given by the Jacobian matrix of the deformation gradient, $\rho_{0}$ the initial density, $v_{i}$ the velocity of the fluid particles at position $x_{i}$ , $\sigma_{ij}$ the Cauchy stress tensor, and $f_{i}$ the fluid forces per unit volume.

In the second Eulerian step, also called advection step, the mesh was remapped to its initial Eulerian position and an advection algorithm was used to calculate the conservation variables. The following transport equations were solved with initial conditions from the solution of the Lagrangian step at the same time (Marom, 2015):

$$\begin{array}{c} \frac{\partial \phi }{\partial t}+\left(v-v_{m}\right)\cdot \nabla \phi =0 \end{array}$$ (4)

Where ϕ is the conservation variable. The coupling of structure and fluid was realized by means of a penalty-based approach, where the problem was regarded as a spring system. The spring was connected to a structure node and a fluid particle, and thus penalty forces proportional to the penetration depth and stiffness coefficient were applied. The coupling force of the fluid particle was then distributed to surrounding fluid nodes using shape functions (Nobari, 2012). The FSI analysis was performed with a time step of 0.01 s and 20 iterations per step. Related equations are as follow:

$$\begin{array}{c} F=k\cdot d \end{array}$$ (5)

$$\begin{array}{c} F_{s}=-F \end{array}$$ (6)

$$\begin{array}{c} F_{f}^{i}=N_{i}\cdot F \end{array}$$ (7)

Where F is the coupling force, k the stiffness coefficient, d the penetration depth, $F_{\text{s}}$ the force at the corresponding structure node, $F_{\text{f}}^{i}$ the force at the surrounding fluid node i , $N_{i}$ the shape function at node i .

The FSI analysis of this study includes the valve opening and closing due to hemodynamics under pulsatile load after three personalized heart valve stent devices were implanted into the aortic valve. The inner wall of the aorta is simplified and is shown as the wall of the fluid domain in the computational model of fluid mechanics. The calculation cost and structural size were measured, the mesh division process was repeatedly adjusted, and the division size was controlled within 0.6 mm.

**FIGURE 7:**
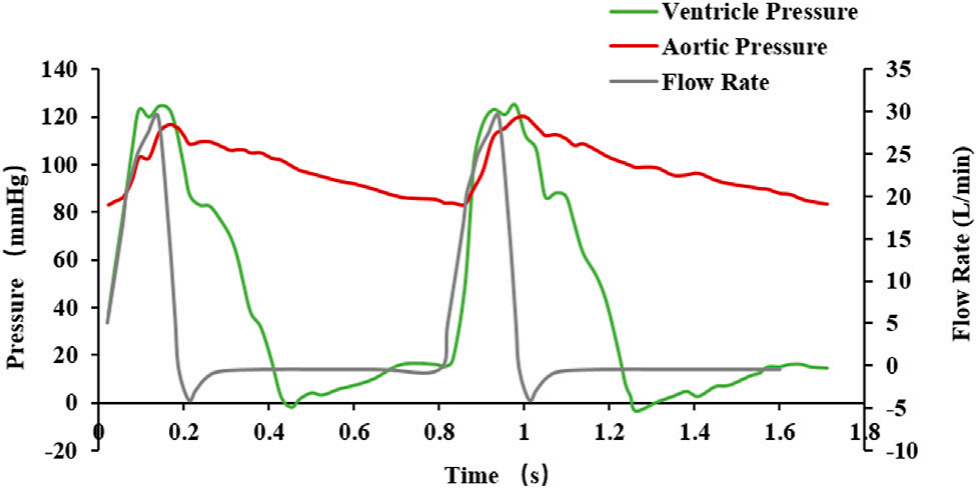
Fluctuating load boundary curve.

For the boundary conditions of the fluid analysis, which relate to the data of a previous study (Sodhani et al., 2018), pressure inlet and pressure outlet conditions under the pulsation cycle load were selected. The boundary condition data are shown in Figure 7, and two pulsation cycles were calculated. In the analysis, blood was considered an incompressible fluid with a density of 1,060 kg/m^3^ and a viscosity of 0.004 Pa s. The turbulence was neglected and the flow was assumed to be laminar. Because the diameter of the blood vessel was larger than 1 mm, the blood was considered to be a Newtonian fluid (Aenis et al., 1997) and the pulsatile loading of the blood was assessed for transient flow analysis. For the mesh generation of the discrete model of the fluid domain calculation, the number of fluid domain elements was 253,748, and the number of nodes was 371,526.

## Result

### Aortic Valve Dynamics

**FIGURE 8:**
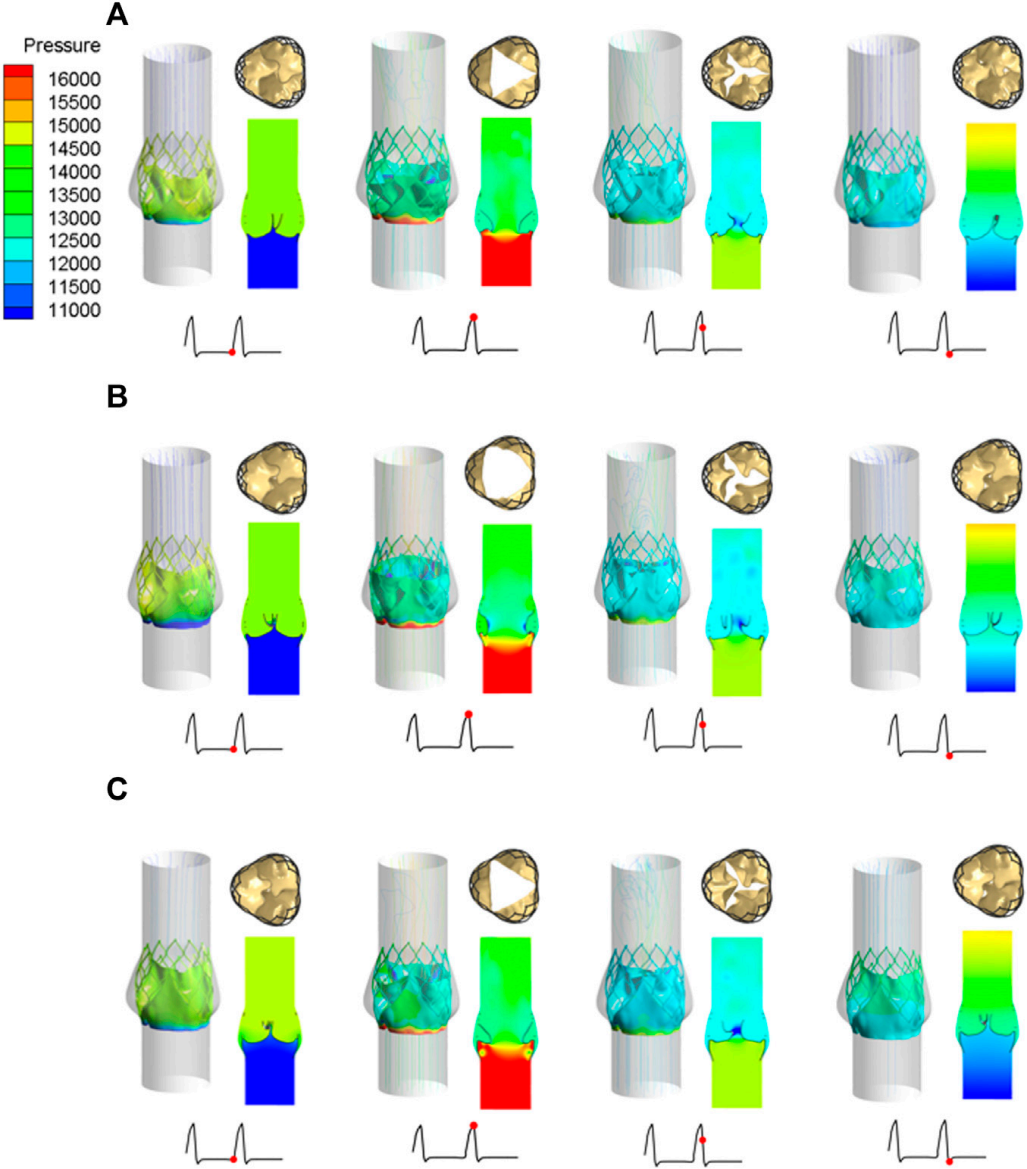
Leaflet opening and closing states and pressure distribution diagrams at four different flow stages, **(A)** valve stent model A, **(B)** valve stent model B, **(C)** valve stent model C.

Under the same boundary conditions, the motion states of the leaflets of the three valve stents were analyzed and compared. Figure 8 shows the opening and closing states of the leaflets and the pressure distributions at four different flow stages. The four flow phases were late diastole (0.8 s) of the first pulsatile cycle, peak systolic phase (0.93 s) of the second pulsatile cycle, maximum deceleration (0.96 s), and early diastole (1.01 s). The flow field pressure assessed the motion state of the valve leaflets. After the three valve stents were implanted, the pressure distributions in the flow field were similar. In the late diastole, the outlet pressure was much greater than the inlet pressure, and the three valve leaflets were in a closed state. At 0.93 s, the inlet pressure was greater than the outlet pressure by about 20 mmHg, and the three leaflets were in a fully open state and near to the maximum opening state. After about 0.03 s, the valve leaflets entered a closed state and remained in a closed state throughout the diastolic period.

During peak systole, the cross-sections of the leaflet openings also showed very different leaflet motion profiles. The opening area of the valve stent was directly calculated according to the shape of the deformed valve leaflet (Figure 8). The edges of leaflet models A and C should fit along the metal frame structure of the stent to the greatest extent possible. In the vicinity of the implanted sinus, due to the dual effects of sinus shape extrusion and blood flow impact, these two leaflet structures appeared as obvious triangular openings. Between the two, the morphological structure with greater curvature at the bottom of the leaflet, that is, the opening area of model C (175.779 mm^2^), was larger than that of model A (134.768 mm^2^), which is consistent with the results of Ghosh et al. (2018). Among the three models, leaflet model B had the largest opening area, of 243.668 mm^2^, and its area was about 1.8 times that of model A, and about 1.4 times that of model C.

**FIGURE 9:**
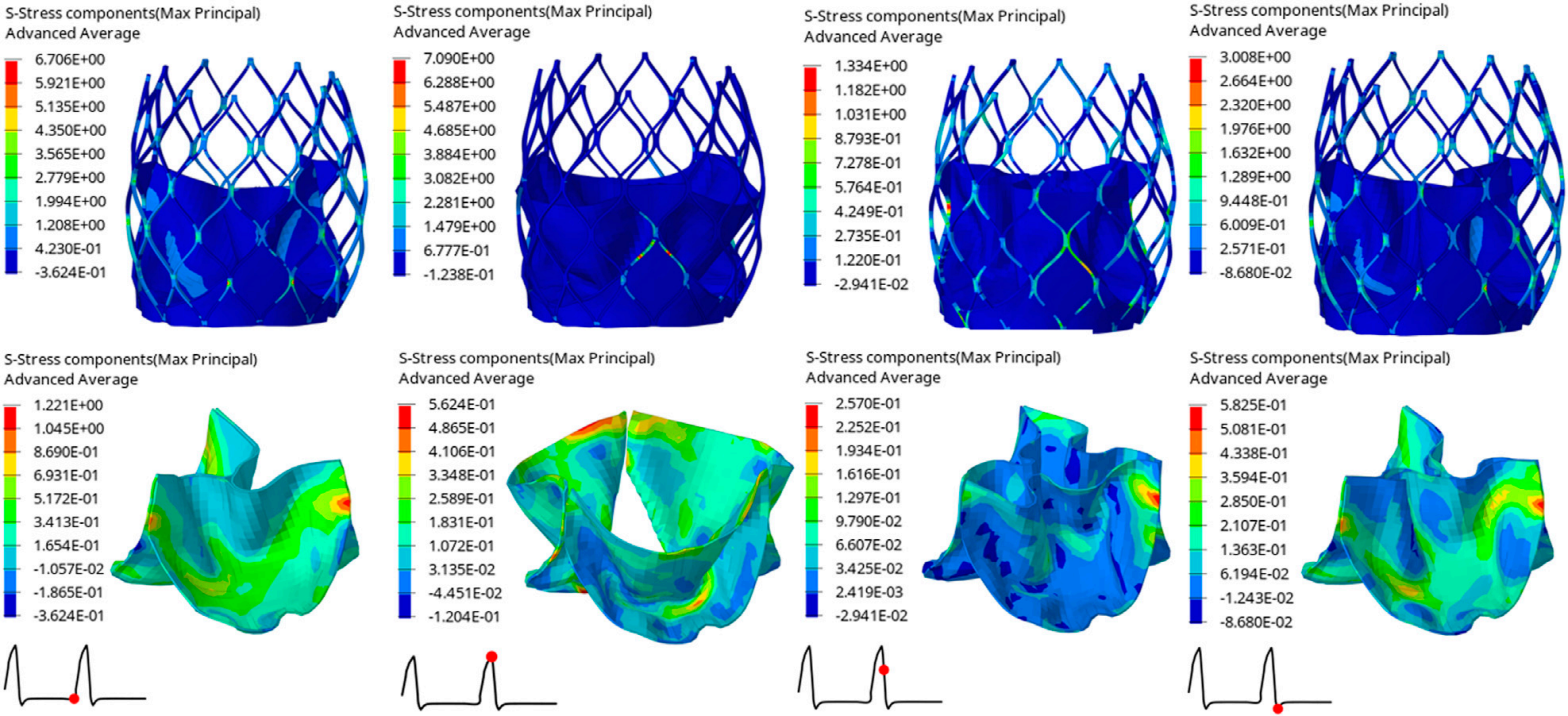
Maximum principal stress distribution of valve stent and valve leaflet of model A.

**FIGURE 10:**
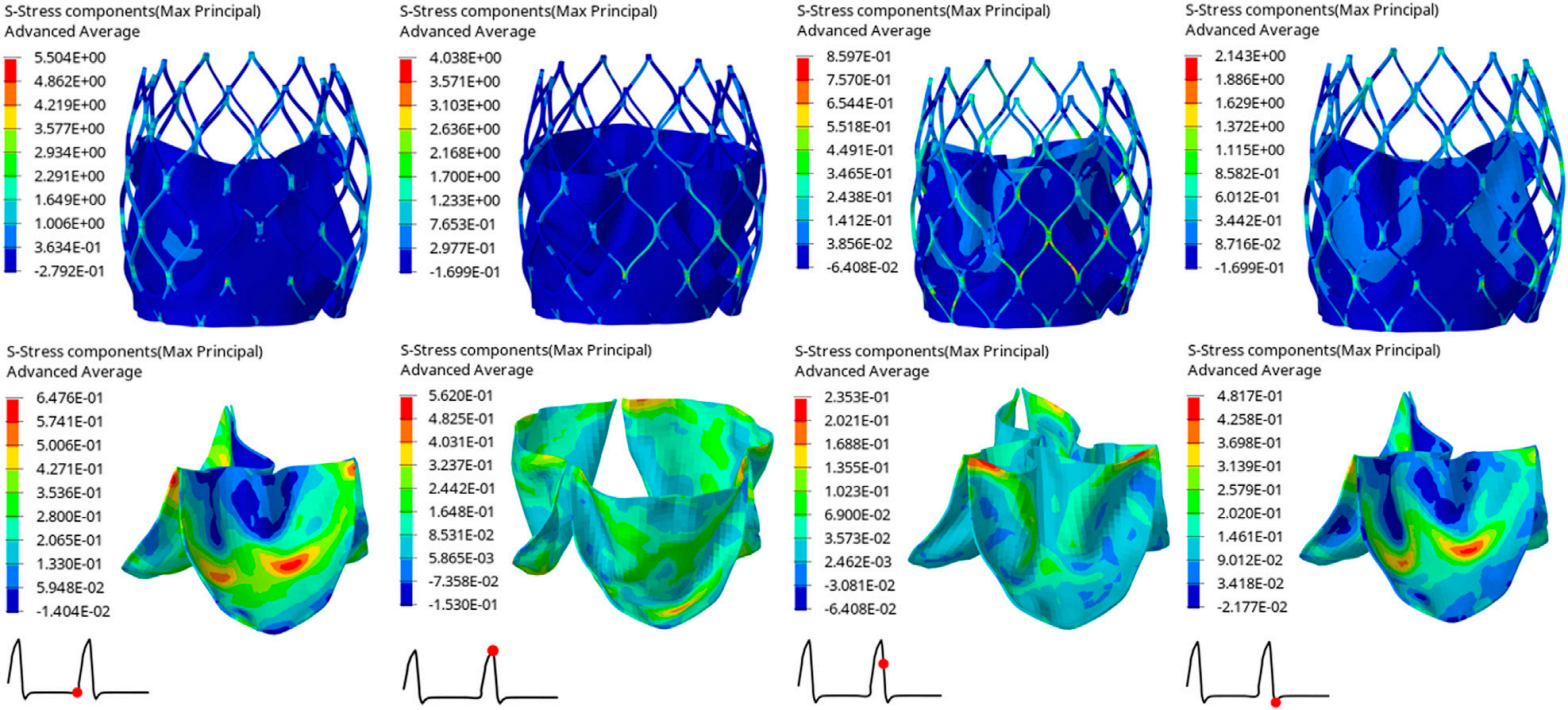
Maximum principal stress distribution of valve stent and valve leaflet of model B.

**FIGURE 11:**
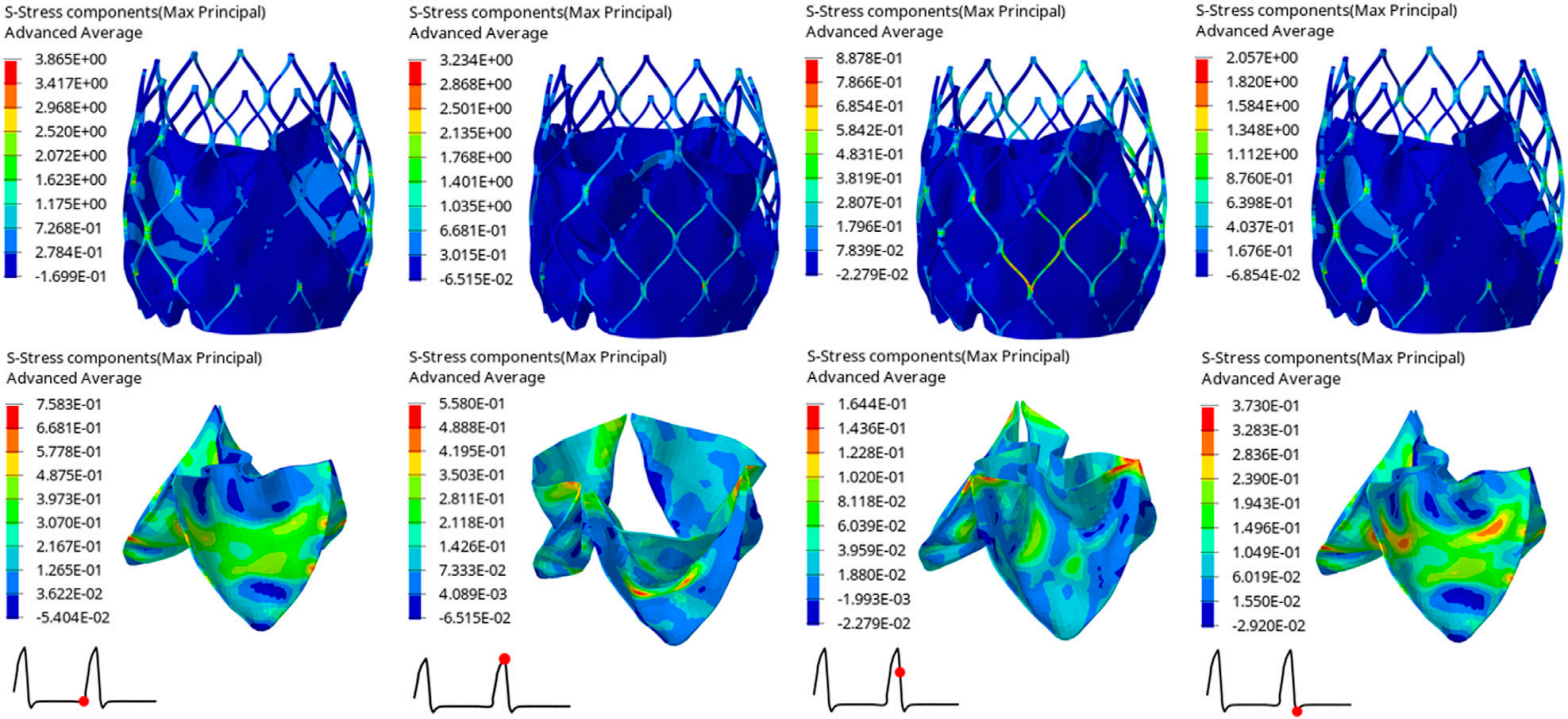
Maximum principal stress distribution of valve stent and valve leaflet of model C.

The structural dynamics of the valve leaflets were compared according to the maximum principal stress and strain values. Figures 9–11 shows the valve stent structure and the maximum principal stress distribution of the valve leaflets. The distribution of the maximum principal stress and strain of the valve leaflets under different loads showed a relationship with the flow pulsation cycle stage and mechanical properties. Due to the extrusion of the stent and valve leaflets by the sinus structure during implantation, the three valve leaflets were not completely consistent in structure. Under the influence of blood flow, the force shape differed slightly, but the stress-strain distribution trend of the three leaflets in the same structure was basically the same. Table 3 shows the maximum principal stress of the valve leaflet and valve stent, and the logarithmic strain (LE) of the valve leaflet. In the middle and late diastole and peak systolic period, both the valve leaflets and the stent had higher stress and strain values, and the stress on the valve stent structure of model A was much greater than that in the other two models (Table 3). The stress and strain were relatively similar between models. The stress on the valve of model A was relatively large, which almost always occurred at the junction of the valve with the stent and skirt, and was close to the outlet end. The maximum stress on the leaflets of models B and C was inside the leaflets, and this large stress was affected by the curvature of the structure when the leaflets were opened and closed.

**TABLE 3:** The maximum principal stress of the valve leaflet and valve stent, the LE of valve leaflet.

	Time(s)	Model A	Model B	Model C
Max. principal stress of valve leaflet (MPa)	0.80	1.2210	0.6476	0.7583
	0.93	0.5624	0.5620	0.5580
	0.96	0.2570	0.2353	0.1644
	1.01	0.5825	0.4817	0.3730
Max. principal stress of valve stent (MPa)	0.80	6.7060	5.5040	3.8650
	0.93	7.0900	4.0380	3.2340
	0.96	1.3340	0.8597	0.8878
	1.01	3.0080	2.1430	2.0570
The LE of valve leaflet	0.80	0.1167	0.0754	0.0776
	0.93	0.1733	0.1101	0.1081
	0.96	0.0239	0.0273	0.0164
	1.01	0.0559	0.0511	0.0469

### Hemodynamic Effects of the Three Valve Stents

**FIGURE 12:**
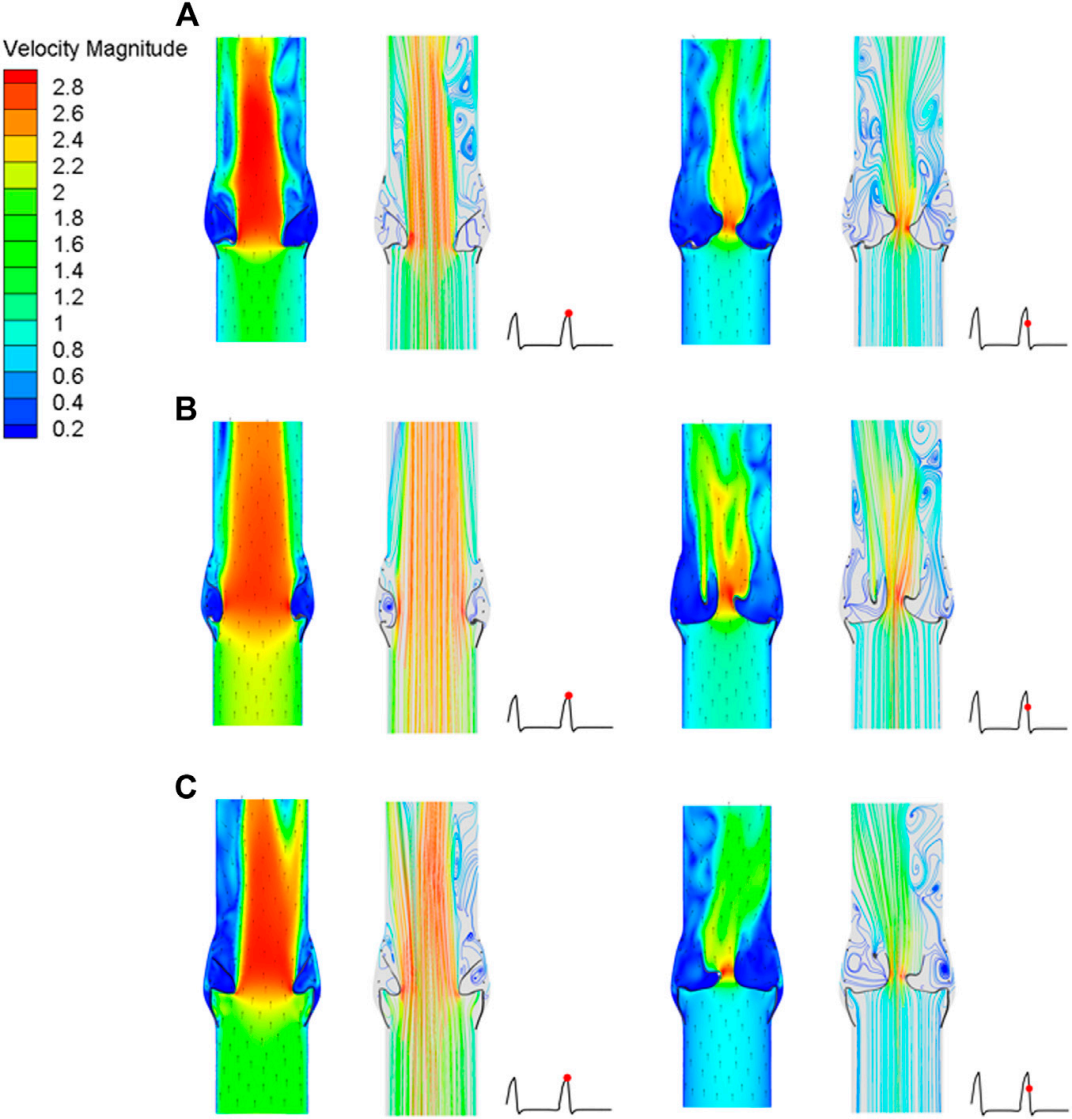
Cross-sectional flow velocity distribution in two flow stages, **(A)** valve stent model A, **(B)** valve stent model B, **(C)** valve stent model C.

After implantation of the three valve stents, the flow fields had similar pressure distributions and blood flow characteristics, but the peak velocities were different. The valve leaflet models A and C had a narrower central jet at peak contraction. The section perpendicular to the axial direction at the maximum convex point of the sinus surface was taken, and the flow rate of the three models at this section was calculated. The maximum flow rate of model A was 337.68 ml/s, that of model B was 536.31 ml/s, and that of model C was 439.02 ml/s; the ejection flow of model B was about 1.6 times that of model A, and about 1.2 times that of model C. The effects of leaflet opening and closing on the flow field were compared during the peak contraction period (0.93 s) and to the maximum deceleration period (0.96 s) of the three models (Figure 12). The velocity streamline diagram in Figure 12 shows the state of the vortex in the flow field when the valve leaflets opened and closed. Model B only had relatively regular counter-rotating vortices in the sinus cavity, while the other two models were close to the outlet of the blood vessel during the peak systolic period. Except model A, the other two models produced multiple vortices, which also affected the opening and closing state of the valve leaflets.

## Discussion

Millions of patients are diagnosed with aortic valve disease every year. The incidence of aortic valve disease caused by degenerative aortic valve changes is up to 10% in the elderly. With many country’s demographics aging, the proportion of populations with aortic valve disease is increasing. Aortic valve diseases are mainly divided into two types: aortic stenosis and aortic insufficiency (Marom, 2015). With the rapid development of modern medicine, most patients’ quality of life can be improved through surgical procedures and minimally invasive interventions. TAVR is a minimally invasive treatment for patients with high-risk aortic diseases. With the development in recent years of interventional therapy technologies, TAVR is used more and more in clinics, but many important factors still need to be studied, especially in the context of blood hydrodynamics. TAVR is mainly used to improve the blood flow through the aortic valve, which involves the FSI at the aortic valve, valve stents and blood flow (Travaglino et al., 2020). A powerful tool to study this problem is numerical analysis. Through numerical analysis, we can realize hemodynamic characteristics that cannot be explored through experiments, simulate the situation after valve stent implantation, and preliminarily complete preoperative evaluation so as to find the best clinical treatment scheme.

Multiple approaches have been applied to the research of FSI analysis technology, including ALE (Nguyen et al., 2011; Cao, 2016; Borowski et al., 2018), “operator split” Lagrangian-Euler method (Luraghi et al., 2019; Pasta et al., 2020), the immersed boundary method (IBM) (Lemmon and Yoganathan, 2000; Jendoubi et al., 2014; Kallemov et al., 2016), and the curvilinear immersed boundary (CURVIB) method (Ge and Sotiropoulos, 2007; Borazjani et al., 2008). Each of these technical methods have their own specific characteristics. When considered in combination with the results of previous studies, insightful research results have been achieved using these methods, which supports the development of the FSI method to study aortic valve disease and its treatment devices. In the present study, the “operator split” Lagrangian-Euler method was chosen to study the relationship between valve stent structure design and flow field. This method solves the conservation equation in two steps: the Lagrangian equation and the Eulerian equation. In the “operator split” Lagrangian-Euler method, the influence of the moving structure is transmitted to the fluid through structural forces, and the advection algorithm is used to couple the structural and fluid domains. The main objective of this study was to use the FSI method, which is based on computational fluid dynamics, to study the mechanical and fluid properties of different valve stent structural designs after aortic valve implantation. Through such basic research, the relationship between structural design and blood can be understood.

The stent material used in this study was nickel titanium alloy, which is a shape memory alloy. When the valve stent is transported to the lesion position, it will self-expanding after release. Using the memory characteristics of the nickel titanium alloy material, after stent implantation, its shape can change adaptively with the change of aortic valve structure, which can enhance the fit between stent and aortic valve, prevent valve stent displacement, and reduce the probability of leakage. Although the self-expanding valve stents have been significantly improved, for some patients with valve diseases the application of TAVR may not achieve good therapeutic effects, and the stability of TAVR and the scope of surgical indications still need to be improved. Valve stent dislocation is a rare but serious complication after TAVR. If it is not treated in time, it seriously affects prognosis. For such patients, it is necessary to improve the stability of self-expanding valve stents through design improvements, in turn to improve the success rate of TAVR implantation. In this paper, simulations of the valve stent implantation process are realized, which is of great significance for the study of the mechanical characteristics between the valve stent and aortic valve tissue, and can be used to evaluate the fit level between them.

With the progress of technology, indications of TAVR have increased. In addition, the age of the target group has decreased. Complications after TAVR have been attended to, especially perivalvular leakage, which directly affects the medium- and long-term life quality of patients after TAVR (Mylotte et al., 2015; Ando et al., 2016; Siemieniuk et al., 2016). In the current study, three personalized stent systems were established to evaluate the kinematic characteristics of the three stent-valve leaflets and their effects on the flow field. The complex three-dimensional flow field characteristics in the valve region were simulated by a fluid-structure interaction (FSI) method. In this study, the valve opening morphology, opening area, stress and strain, hemodynamic flow field distribution, and pressure distribution of three heart valve devices with the same valve and blood contact area were compared. It can be seen from the analysis results that the leaflet shape had a significant impact on the overall performance of the stent, which means that better hemodynamic performance of TAVR will improve that performance. The simulation of three models verified the repeatability and effectiveness of the FSI method, which can be applied to the design of aortic valve stent devices. FSI was used to analyze the TAVR of the whole heart pulsation cycle and to evaluate the stent performance and hemodynamics completely, aiding in the design of better aortic valve stent devices.

This study has the following limitations: 1) The ideal aortic valve model was used in this paper, and the pathological valve model was not applied to FSI; 2) In the simulation analysis, the aortic valve was not fully considered in the process of valve stent implantation and FSI analysis; 3) Only one stent structure was designed, and the influence of different stent structures on mechanical properties was not fully investigated. 4) Turbulence models were not included in the analysis of the current study. The blood fluid model used in this study is considered to be an incompressible Newtonian fluid, and laminar flow model and actual *in vivo* blood flow pulsation patterns are used for transient flow analysis. In subsequent research, the study should be combined with the patient’s pathological aortic model, and various clinical factors should be gradually added into the study. The turbulence model should be considered in order to achieve a better technology progress. In addition, this calculation model and analysis method requires a large amount of computation, so it is necessary to try different CFD models and FSI models to achieve higher computational efficiency. Greater investment in computing power is also required to achieve higher computational capacity. The results of this study can underpin key breakthroughs in product design, and provide important theoretical support and technical guidance for clinical research.

## Conclusion

The FSI analysis results of three personalized stent systems was compared in the current study. The main difference in the leaflet kinematics was that, during systole, the leaflets were pushed outward by a strong jet stream, with models A and C both forming triangular-like openings while model B leaflets formed nearly circular openings. The different opening shapes made the opening area and instantaneous flow rate of model B larger than those of the other two models. The magnitudes of structural stress and strain provided insight into potential areas where leaflets and stents may fail. The stress concentration of the leaflet of model A mainly occurred near the attachment point of the stent, and the outlet end received the highest stress and strain values, with the maximum value beings greater than that of the other two models, which may cause damage to the leaflet at the connection with the stent and the skirt. Where the stress and strain of models B and C were the largest, this was mostly caused by a change in valve shape and a large change in the curvature of the curved structure. Comparing the effects of model B and the two models, all had a more ideal vortex shape on the flow field in the systolic period. In the existing valve stent design, greater attention was paid to the mechanical properties and reliability of the stent structure, but the influence of valve shape was ignored. In this study, three valve leaflet shapes were designed for the same stent frame structure. The fluid-structure coupling calculation showed that the impact of the valve leaflet shape on the overall performance of the stent was important, and superior hemodynamic effects could also improve the stent performance.

## Data Availability

The original contributions presented in the study are included in the article/Supplementary Material, further inquiries can be directed to the corresponding author.
